# Synthesis and surface grafting of a β-cyclodextrin dimer facilitating cooperative inclusion of 2,6-ANS

**DOI:** 10.3762/bjoc.11.58

**Published:** 2015-04-21

**Authors:** Lars W Städe, Thorbjørn T Nielsen, Laurent Duroux, Reinhard Wimmer, Kyoko Shimizu, Kim L Larsen

**Affiliations:** 1 Department of Biotechnology, Chemistry and Environmental Engineering, Aalborg University, Frederik Bajers Vej 7H, DK-9220 Aalborg East, Denmark; 2 Department of Chemistry, Aarhus University, Langelandsgade 140, DK-8000 Aarhus, Denmark

**Keywords:** 2,6-ANS, β-cyclodextrin dimer, silicon dioxide, surface grafting, total internal reflection fluorescence spectroscopy

## Abstract

A novel β-cyclodextrin (β-CD) dimer was synthesized and surface-grafted by click chemistry onto azide-functionalized quartz surfaces in order to introduce the cooperative features of the β-CD dimer to solid surfaces. Using NMR and fluorescence spectroscopy, it is shown that the free β-CD dimer forms a 1:1 complex with the fluorescent guest molecule, 2-anilinonaphthalene-6-sulfonic acid (otherwise known not to form 1:2 complexes with parent β-CD), with an apparent association constant of 7300 M^−1^. Further, it is shown using total internal reflection fluorescence spectroscopy that the inclusion of the fluorescent guest into both cavities of the β-CD dimer is maintained when grafted onto a solid surface.

## Introduction

Since the initial reports [1–4] of the cooperative effects exerted by β-cyclodextrin (β-CD) dimers, they have been suggested for a wide range of applications, for example, for catalysis [5–6] and photochemistry [7–8], as synthetic enzymes [5] and in general to obtain improved binding affinity (as compared to parent β-CD) for the inclusion of lipophilic molecules [5,9]. The extensive research on the topic has generated a vast number of different β-CD-dimer constructs and methods for the synthesis thereof. Of these methods, synthesis by click chemistry (and specifically the copper(I)-catalyzed azide−alkyne cycloaddition (CuAAC)) is very attractive [10–11]. CuAAC is known to be selective, proceed fast and produce a high yield under mild conditions. Further, the formed triazole is stable against oxidation, reduction and hydrolysis [10]. Concerning the design of β-CD dimers, it was recently demonstrated that the choice of linker is an important parameter in order to fully access both cavities [12–13]. Specifically, it was demonstrated that long and/or hydrophobic linkers may induce an inversion process, resulting in self-inclusion of the linker in one of the cavities, thereby limiting accessibility for inclusion-complex formation [13].

The research presented here aims to bring the cooperative effects of β-CD dimers to solid silicon dioxide surfaces. Modification of these surfaces allows the introduction of the extraordinary binding affinity and selectivity of CD dimers to silicon wafer technology and allows the potential cooperative effects to be exploited within chromatographic applications by grafting to silica gels. Further, silicon dioxide surfaces in the form of quartz and glass allow the detection and monitoring of binding events by optical techniques such as fluorescence spectroscopy and microscopy.

Numerous methods have already been reported for the functionalization of planar silicon dioxide surfaces with monomeric β-CD for supramolecular inclusion of appropriate guest molecules [14]. Further, it has been demonstrated that such surfaces may serve as a multivalent receptor [15–20] and thereby can potentially exert effects similar to those of a β-CD dimer. However, these effects will be dependent on and limited by the β-CD density, preferential conformations and, to a certain extent, steric limitations related to the linker moiety facilitating the functionalization. Controlled surface grafting with a well-defined β-CD dimer is, on the other hand, expected to address these issues and allow for the preparation of a homogenous surface exhibiting well-defined divalent binding sites.

To meet this objective, a novel method for the facile synthesis of a β-CD dimer was developed in line with the aforementioned strategy of preparation of dimers by CuAAC. In this work, however, 6-monodeoxy-6-monoazido-β-CD (N_3_β-CD) is coupled with tripropargylamine in a 2 to 1 ratio for synthesis of a β-CD dimer with a free alkyne functionality, allowing for subsequent surface grafting onto azide-functionalized quartz surfaces. Host–guest interactions with the fluorescent guest molecule 2-anilinonaphthalene-6-sulfonic acid (2,6-ANS) were investigated by NMR and fluorescence spectroscopy. Further, we probe the complex formation of the surface-grafted β-CD dimer with 2,6-ANS by employing a sensitive surface technique, total internal reflection fluorescence (TIRF) spectroscopy.

## Results and Discussion

### Synthesis of the β-CD dimer

**Scheme 1:**
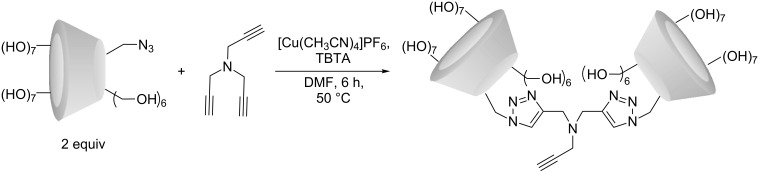
Synthetic route for the synthesis of the β-CD dimer with a free alkyne, allowing for subsequent surface grafting.

**Figure 1:**
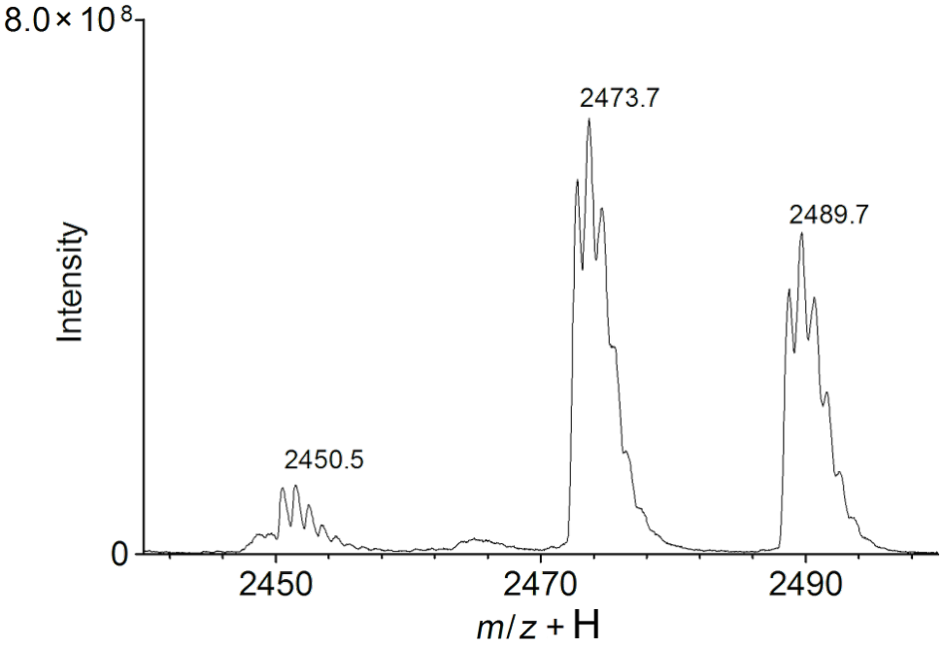
MS spectra (2440–2500 Da) of the purified β-CD dimer.

The synthetic route resulting in the β-CD dimer is depicted in Scheme 1. With tripropargylamine as a scaffold, the resulting structure resembles the β-CD dimer synthesized from dipropynyl ether by Potier et al. [13]. For this structure, it was shown that self-inclusion does not occur, and is thus not considered as an issue here. Two equivalents of N_3_β-CD to tripropargylamine were used in order to favor formation of a dimer over a monomer, based on the quantitative nature of the CuAAC. The formation of a β-CD trimer was not anticipated to be of major concern because of the unfavorable steric interactions upon grafting of a third β-CD onto the β-CD dimer. This was confirmed during purification by flash chromatography by observation of a major peak with a minor peak at the front edge and a smaller shoulder at the trailing edge, corresponding to the trimer and monomer, respectively. The fractions corresponding to the main peak were collected and analyzed by NMR (see spectra in Supporting Information File 1), which confirmed the presence of the expected chemical shifts. The product was further analyzed by MALDI–TOF mass spectrometry for which the dominant contributions in the region of 2400 to 2500 Da (Figure 1) were found. The three peaks observed in this region correspond to the β-CD dimer (2451 Da), sodium (2473 Da) and potassium adducts (2490 Da). In addition, minor fractions corresponding to the triazole-β-CD monomer and β-CD trimer were observed (not shown). In the present context, the occurrence of the monomeric form is of greatest concern; however, from the NMR spectra (Supporting Information File 1) it is evident that the monomer fraction is negligible.

### Inclusion complex with free β-CD dimer in solution

**Figure 2:**
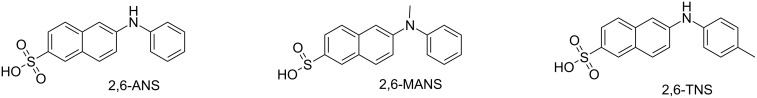
Structure of fluorescent guest molecule, 2,6-ANS, used to probe host–guest interaction and two derivatives thereof with methyl substitutions at the anilino moiety.

**Figure 3:**
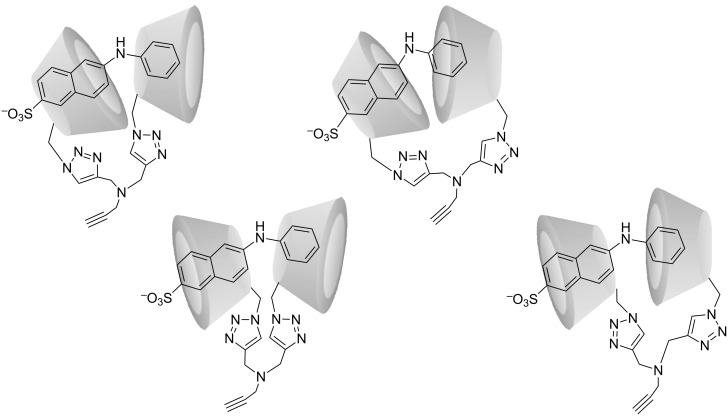
Illustration of potential 1:1 inclusion complexes of the β-CD dimer and 2,6-ANS.

Host–guest interactions with the fluorescent probe 2,6-ANS were evaluated for the β-CD dimer by steady-state fluorescence spectroscopy and compared to that of the parent β-CD and 2,6-ANS. In water, 2,6-ANS displays only weak fluorescence, primarily ascribed to the quenching effect of dipolar water molecules. Upon inclusion of 2,6-ANS into the hydrophobic β-CD cavity, the fluorescence yield increases accompanied by a blue shift of the emission maximum due to the change in polarity (i.e., shielding from aqueous solution) [21–22]. Although the coplanar arrangement of the aromatic rings makes 2,6-ANS an excellent guest molecule, the dimensions of 2,6-ANS exceed the dimensions of the β-CD cavity and, therefore, potentially allow for formation of a 2:1 complex. This behavior has previously been reported for parent β-CD with the naphthalene derivatives, 2-(*N*-methylanilino)naphthalene-6-sulfonic acid (2,6-MANS) and 2-(*p*-toluidinyl)naphthalene-6-sulfonic acid (2,6 TNS) [23]. These derivatives differ from 2,6-ANS only by having a methyl substitution at the aniline moiety (Figure 2). In the relevant study [23], the formation of a stable 2:1 complex was ascribed to the protection of these moieties from the aqueous solution by inclusion in the β-CD cavity in an equatorial approach, resulting in the inclusion of the naphthalene and anilino moieties in each of their respective β-CD cavities. The study [23] also included 2,6-ANS for which only a 1:1 complex with parent β-CD was observed. We speculated that a β-CD dimer prepared with a short spacer would allow for the formation of 1:1 complex (1:2 in terms of β-CD moieties) with molecules bearing extended aromatic rings (such as the 2,6-ANS used here as a model), thereby resulting in higher complex stability compared to that of parent β-CD. The potential conformations for 1:1 complexes of the β-CD dimer with 2,6-ANS are illustrated in Figure 3.

**Figure 4:**
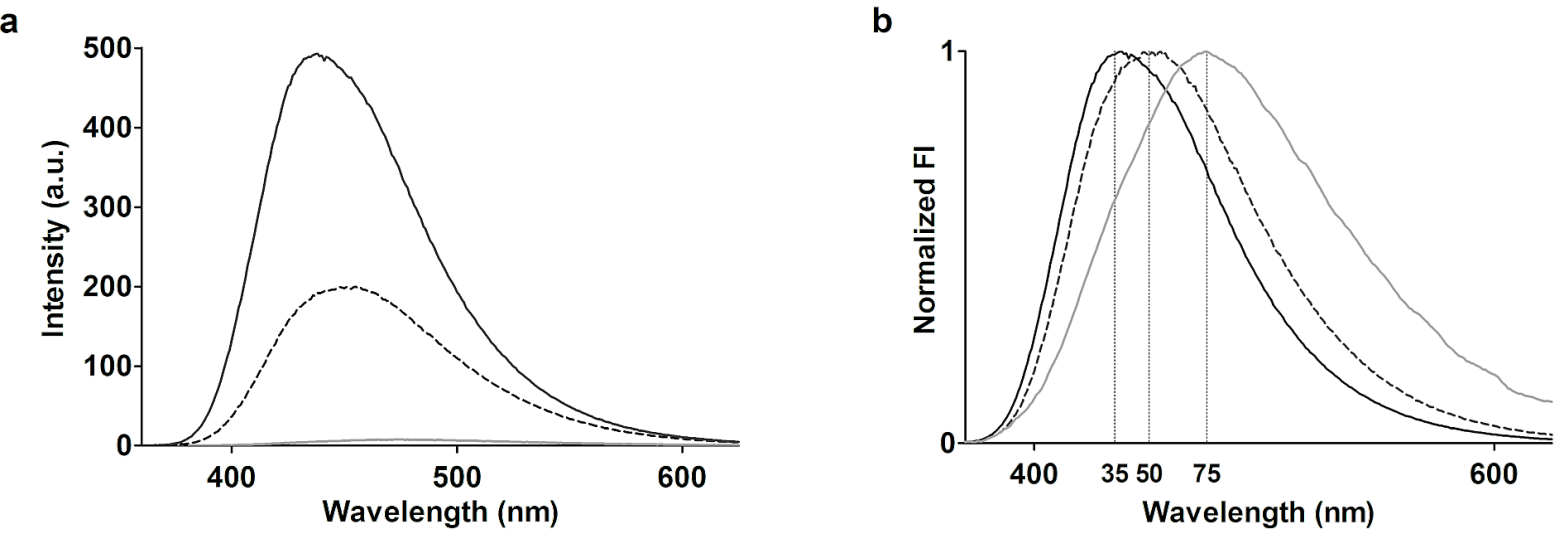
(a) Steady-state fluorescence emission spectra of 50 µM 2,6-ANS in PBS (solid grey, smoothed), in the presence of 4 mM β-CD (black dotted) and in the presence of 2 mM β-CD dimer (plain black) and (b) the corresponding normalized spectra. The emission maxima are in the listed order 475, 450 and 435 nm.

Figure 4a shows the fluorescence emission spectra of 50 µM 2,6-ANS (solid grey) in phosphate-buffered saline (PBS, pH 7.4) and 50 µM 2,6-ANS in PBS with 2 mM β-CD dimer (solid black) and 4 mM parent β-CD (dotted black). As previously mentioned, the emission spectra are affected by the polarity of the environment surrounding the 2,6-ANS, whereby a comparison of the spectra allows for evaluation of the β-CD dimer/2,6-ANS inclusion complex. From Figure 4 it is evident that the 2,6-ANS inclusion complex and the β-CD dimer have a significantly higher fluorescence intensity (FI) as compared to the parent β-CD, despite that the total concentration of β-CD cavities is the same. However, as the FI is sensitive to changes in host concentration (i.e., the number of formed complexes), the accompanying shift in the emission energy maximum serves as a more suitable probe. The extent of the shift in the emission spectra is clearly illustrated in the normalized spectra of the three solutions (Figure 4b): a significant blue shift is observed from 475 nm for free 2,6-ANS to 450 nm and 435 nm for the 2,6-ANS solutions with parent β-CD and the β-CD dimer, respectively. These observations lead to the conclusion that 2,6-ANS experiences a more non-polar environment upon inclusion complex formation with the β-CD dimer. For the inclusion of parent β-CD, it has been previously shown that 2,6-ANS likely enters the cavity in an equatorial approach with inclusion of the naphthalene moiety in the cavity, whereby both the sulfonate and the anilino moiety are solvated [23]. Consequently, the formed complex may be relatively dynamic with the charged sulfonate protruding into the aqueous solution. This is assisted by the hydrophobic anilino moiety that seeks the cavity in competition with the more hydrophobic naphthalene moiety. In the case of the β-CD dimer, the introduction of a fixed second cavity allows for complexation with the anilino moiety. This complex could eventually be further stabilized by hydrogen bonding, involving rim hydroxy groups from both cavities and the amino function.

**Figure 5:**
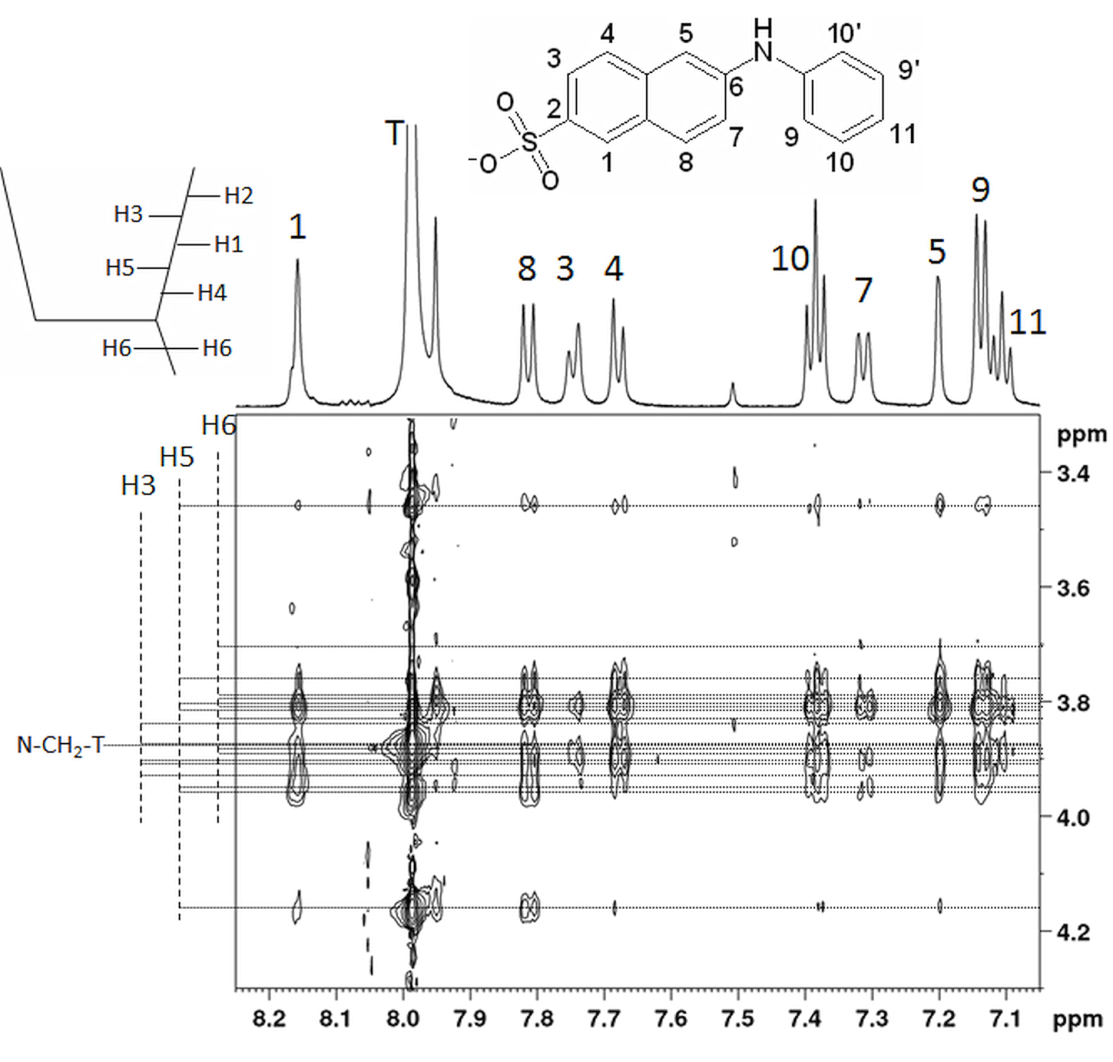
Region of interest of a ROESY (250 ms mixing time) showing cross peaks between the β-CD-dimer and 2,6-ANS. The corresponding region of the ^1^H NMR spectrum of the mixture is shown above the 2D plot. The signal marked “T” is related to the single proton attached to the triazole ring. Dotted, horizontal lines indicate the positions of the β-CD H3, H5 and H6 resonances of the mixture: the lines extending furthest to the left correspond to H3 atoms, lines extending least to the left side correspond to H6, lines of intermediate length belong to resonances of H5. Dashed, vertical lines and their corresponding label also indicate to which atom the resonances belong. The resonance marked “N-CH_2_-T” comes from the CH_2_ group between the triazole moiety and the nitrogen atom connecting the two β-CD units.

The binding mechanism suggested on the basis of the changes in fluorescence emission is confirmed by the NMR ROESY spectrum of the β-CD dimer/2,6-ANS inclusion complex (Figure 5). Due to the lack of symmetry of the substituted β-CD, each of the seven glucose units (constituting the two β-CD units of the dimer) can be found as distinguishable sets of NMR resonances. Although the set of H-3 and H-5 resonances can be distinguished well, the H-6 resonances are distributed in a way that they overlap with both the H-3 and H-5 resonances. This makes an unambiguous and quantitative evaluation of the ROESY spectrum in terms of the inclusion complex structure quite impossible. However, ROESY cross peaks show that both the naphthyl and phenyl ring are included into β-CD cavities. Looking at the naphthyl ring system, the 2,6-ANS hydrogen atoms 1, 4, 5 and 8 yield the strongest cross peaks with nuclei from the β-CD, while H-3 and H-7 yield only weak cross peaks. In the phenyl ring system, H-9, 9’, 10 and 10’ show the strongest interaction with β-CD hydrogen atoms, while H-11 shows only a weak interaction. It was also previously reported that the H atom of a monosubstituted phenyl ring *para* to the substituent yields only weak cross peaks because of its position further away from the cavity walls [24]. All ROESY cross peaks appear strongest for β-CD H-5 and somewhat weaker for β-CD H-3. The fact that the cross peaks for H-5 seem to be the strongest could suggest that the guest molecule enters the β-CD cavity from the narrow rim. However, the linker atom, i.e., the CH_2_ group between the triazole ring and the nitrogen atom connecting the two monomeric subunits, exhibits cross peaks with 2,6-ANS and also overlaps with H-3 resonances. Thus, it is not possible to assess the contribution of cross peaks to H-6 and no concrete conclusions about the inclusion complex structures could be made. No ROESY cross peaks between atoms of 2,6-ANS and β-CD H-2 and H-4 were observed (with one exception: the resonance at 3.46 ppm, these hydrogen atoms do not overlap with H-3 and H-5).

**Figure 6:**
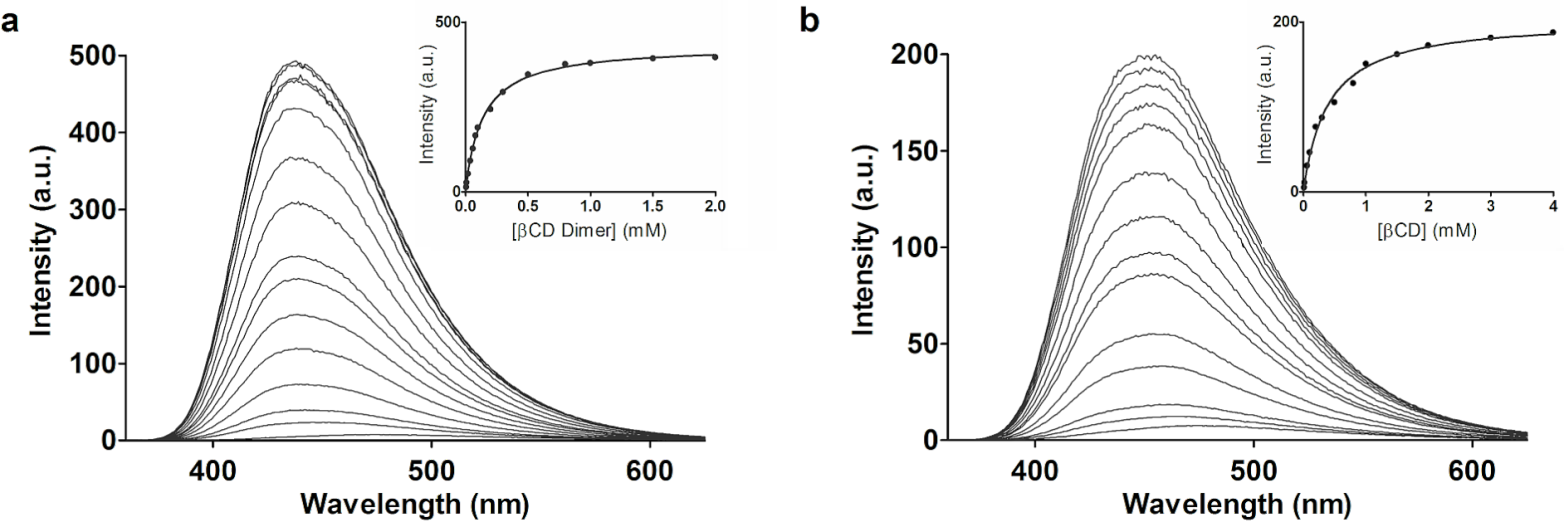
Steady-state fluorescence titration of 2,6-ANS with β-CD dimer and parent β-CD in solution. 2,6-ANS (50 µM) was titrated with 0 to 2 mM β-CD dimer (a) and 0 to 4 mM parent β-CD (b). The FI increases with respect to increasing concentration of β-CD dimer or β-CD. Inserts: Binding isotherms derived from the increase in FI at 420 nm as a function of β-CD dimer or β-CD concentration.

In order to determine the binding affinity and to confirm the 1:1 stoichiometry of the complex, fluorescence titration experiments were conducted. Figure 6 shows the fluorescence spectra of 50 µM 2,6-ANS in PBS recorded at different concentrations of β-CD dimer (Figure 6a) and parent β-CD (Figure 6b) at equilibrium. The apparent association constant (*K*_a_) was determined by plotting the variation in FI at 420 nm as a function of the β-CD dimer (Figure 6a, insert) and parent β-CD (Figure 6b, insert) concentration. The data was fitted to a binding isotherm by nonlinear regression, assuming a 1:1 stoichiometry, and yielded a *K*_a_ of 7300 M^−1^ and 2550 M^−1^ for the β-CD dimer and parent β-CD, respectively. The *K*_a_ obtained for the parent β-CD is in agreement with earlier findings [23]. The approximately 3-fold increase in *K*_a_ reveals a notably stronger binding of 2,6-ANS to the β-CD dimer, as compared to the parent β-CD. Further, the goodness of fit confirms the 1:1 stoichiometry (R^2^ = 0.998). The steady-state fluorescence titration experiment thereby appears to confirm the suggested binding mode, that is, the inclusion of 2,6-ANS into both cavities of the β-CD dimer.

### Surface grafting

**Scheme 2:**
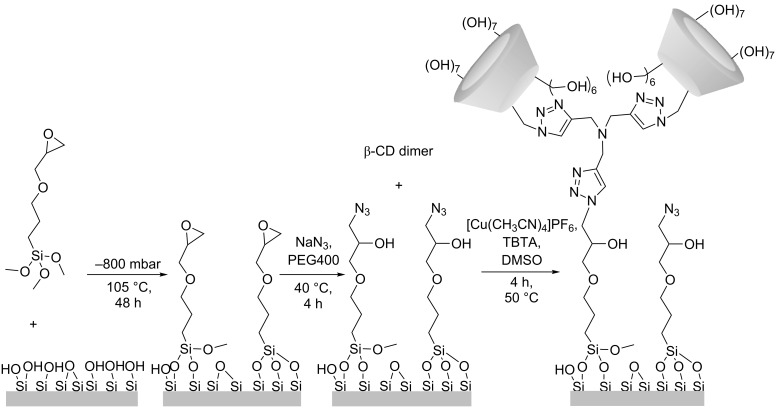
Synthetic route for the activation of silicon dioxide surfaces and the grafting of the β-CD dimer through the free alkyne using CuAAC.

The β-CD dimer was grafted onto quartz slides activated for CuAAC using an in-house method for the preparation of azidosilane monolayers (unpublished results) involving vapor deposition of 3-glycidoxypropyltrimethoxysilane (GPTMS) and subsequent ring opening of the epoxide by sodium azide in PEG400 (Scheme 2). In addition to surface grafting of the β-CD dimer, an azide-functionalized quartz slide was grafted with propargyl alcohol (PA, not shown), by CuAACas well, in order to probe changes in 2,6-ANS fluorescence related to interactions with the silane linker.

The change in chemical composition after silanization with GPTMS and grafting of the β-CD dimer by CuAAC was verified by X-ray photoelectron spectroscopy (XPS). Table 1 displays the chemical surface composition of an unmodified, an epoxy-activated, and a β-CD-dimer-grafted quartz surface. In response to both silanization and grafting of the β-CD dimer, the carbon content increases (C 1s, 285.0 eV), while the appearance of nitrogen (N 1s, 399.5 eV) is observed only for the β-CD dimer-modified surface. Furthermore, a decrease in oxygen (O 1s, 533.0 eV) and silicon (Si 2p, 103.5 eV) content is observed, which can be attributed to an increased shielding of the underlying quartz surface.

**Table 1:** Surface composition of unmodified, epoxy-activated and β-CD-dimer-grafted quartz.^a^

Surface	Atomic percentages			
	C 1s	O 1s	N 1s	Si 2p

Quartz	7.2	64.0	0	28.8
GPTMS	13.2	59.3	0	27.7
β-CD dimer	16.2	57.4	0.5	25.9

^a^Binding energy for C 1s is used as a reference binding energy.

### Evaluation of inclusion complex of surface-grafted β-CD dimer

The inclusion-complex formation of the surface-grafted β-CD dimer with 2,6-ANS was probed by fluorescence spectroscopy by employing the sensitive surface technique, TIRF spectroscopy. This technique allows for monitoring of surface binding events within ≈200 nm of an optical transparent substrate.

**Figure 7:**
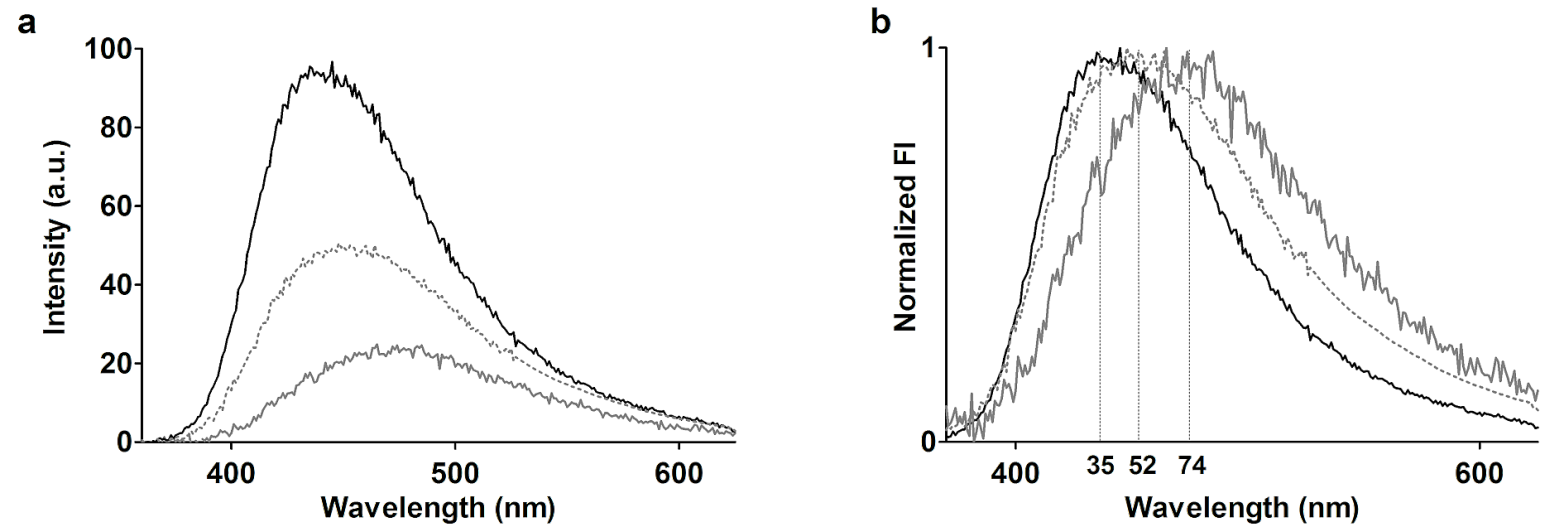
(a) Steady-state TIRF emission spectra of 1 mM 2,6-ANS in PBS, recorded on a bare quartz slide (solid grey), and azide-functionalized quartz slides grafted with PA (dotted grey) and the β-CD dimer (solid black). The corresponding normalized spectra are given in (b). The emission maxima are listed in the order 474, 452 and 435 nm.

Figure 7a shows the fluorescence emission spectra after injection of 1 mM 2,6-ANS in PBS (grey) on a bare quartz slide and quartz slides grafted with PA (dotted black) and the β-CD dimer (plain black) by CuAAC, and Figure 7b shows the corresponding normalized spectra. While the emission spectral envelope obtained on the bare slide, as expected, resembles that of free 2,6-ANS in solution (Figure 4), the FI appears relatively higher and is accompanied by a blue shift to 452 nm for the control slide grafted with PA. These changes are attributed to the hydrophobic environment of the linker. The spectra recorded for the β-CD dimer resembles that of the free dimer; however, the peak appears broader, which is attributed to a possible contribution from the linker and the inversion of the host–guest concentration ratio as compared to fluorescence measurements of the free β-CD dimer in solution. In solution, the concentration of the β-CD dimer is kept in excess of the 2,6-ANS. On the surface, the fixed concentration of the β-CD dimer is lower relative to the 2,6-ANS concentration and results in a high fraction of unbound 2,6-ANS, with an emission spectrum that overlaps with that of the bound 2,6-ANS. However, in light of the observed blue shift of the emission energy of 2,6-ANS (Figure 7b) on the β-CD dimer slide (solid black) compared to the bare slide (solid grey) and the PA slide (dotted grey), and the fact that the emission maximum at 435 nm is identical to that obtained with the free β-CD dimer in solution (Figure 4, solid black), it is concluded that the β-CD dimer is grafted and that the binding mode involving inclusion into both cavities is preserved.

Finally, it should be noted that titration experiments corresponding to those conducted in solution were not conducted as the grafting of the β-CD dimer implies that 2,6-ANS is used as titrant. This approach is unsuitable due to the low solubility of 2,6-ANS in aqueous solution (<1 mM), and more importantly, due to the fact that the quantification parameter is being changed and that this change is not linear as 2,6-ANS displays a self-quenching effect at concentrations above 300 µM (titration series on bare quartz in Supporting Information File 1). This effect is presumably the result of self-aggregation/stacking or self-quenching due to the high concentration of 2,6-ANS.

## Conclusion

A novel method for the facile synthesis of a β-CD dimer containing an alkyne for surface grafting by CuAAC was developed in order to introduce the unique properties of β-CD dimers to solid surfaces. It is shown by ROESY NMR and conventional fluorescence spectroscopy that the prepared β-CD dimer forms a 1:1 complex with the fluorescent β-CD guest, 2,6-ANS. The fact that this guest molecule is included into both of the β-CD cavities of the dimer is extraordinary since it is otherwise known not to form 1:2 complexes with parent β-CD. Further, it is demonstrated that the β-CD dimer can be surface-grafted onto solid silicon dioxide surfaces, and by employing TIRF spectroscopy it was shown that the β-CD dimer maintains its inclusion complex properties when grafted onto a solid surface.

## Experimental

**Materials:** Quartz slides (25 × 38 × 1 mm) were purchased from TIRF Labs, Inc. (Morrisville, NC, USA). β-CD (Wacker-Chemie, Burghausen, Germany) was dried for 24 h at 110 °C under vacuum prior to use. 2,6-ANS (Invitrogen, Oregon, USA), PEG400 and DMSO (VWR International, Fontenay-sous-Bois, France) were used as received. 6-*O*-Monotosyl-β-CD (TsOβ-CD) (Nielsen et al. [25]), N_3_β-CD (Nielsen et al. [25]) and (tris(benzyltriazolylmethyl)amine (TBTA) (Chan et al. [26]) were prepared according to literature protocols. All other chemicals were obtained from Aldrich (Steinheim, Germany) and used as received.

**General procedures:** MALDI–TOF mass spectrometry was performed on a Reflex III (Bruker Daltonics, Bremen, Germany) and automated flash chromatography was performed on a Grace Davidson Discovery Science Revelaris system. NMR spectra were recorded at 298.1 K on a Bruker AVIII-600 MHz instrument equipped with a TCI (H/C/N) probe. XPS analysis was achieved using a Kratos Axis Ultra-DLD spectrometer (Kratos Analytical, Ltd.) and spectral processing was performed using CasaXPS v. 2.3.15 (Casa Software, Ltd.). TIRF spectroscopy was performed on a Varian Cary Eclipse fluorescence spectrophotometer (Varian, Inc.) equipped with a TIRF flow system (TA1004) mounted with a temperature-controlled flow cell and interfaced with digital fluidics SmartFlow TF1005 (TIRF Labs, Inc.).

**Synthesis of β-CD dimer:** N_3_β-CD (2 g, 1.72 mmol), tripropargylamine (115.3 mg, 0.86 mmol), and TBTA (25.1 mg, 0.047 mmol) in 20 mL DMF solutions were degassed by bubbling argon through the solution. Cu(CH_3_CN)_4_PF_6_ (16 mg, 0.043 mmol) was added in one portion and the solution was further degassed for 5 min. The temperature was raised to 50 °C and the mixture was stirred for 6 h under argon. The mixture was poured into 300 mL acetone and the precipitated crude product was filtered off before being recrystallized by dissolving it in 20 mL water and precipitating it in 200 mL acetone. After filtration and preliminary drying, the product was dissolved in 10 mL water and purified on a Grace Revaleris flash system with a 120 g C18 column using a linear water/acetonitrile gradient (0–15%). The appropriate fractions were combined and lyophilized yielding 681 mg of the product as white powder (32%). ^1^H NMR (D_2_O) δ (ppm) 8.04 (s, 2H), 5.18 (d, 2H), 5.12–4.95 (m, 14H), 4.65 (m, 2H), 4.23 (t, 2H), 4.06–3.45 (m, 80H), 3.33 (s, 2H), 3.22 (d, 2H), 2.94 (d, 2H), 2.79 (t, 1H).

**Surface grafting:** Optically transparent silica slides were preactivated with azidosilane monolayers for the CuAAC grafting by vapor deposition of GPTMS (−800 mbar, 48 h, 105 °C) in 400 mM sodium azide in PEG400 [27] for 4 h at 40 °C. Following azidolysis, the substrates were thoroughly rinsed with milli-Q water, sonicated for 5 min in EtOH and dried under a stream of dry nitrogen. The slides were transferred to 50 mL Greiner tubes containing 200 µM β-CD dimer or PA in 30 mL of degassed DMSO, followed by the addition of TBTA (60 µM) and Cu(CH_3_CN)_4_PF_6_ (50 µM) under a nitrogen atmosphere. The reaction tubes were placed on an orbital shaker (500 rpm) and left for 5 h at 50 °C at which point the reaction was repeated for the β-CD dimer slide, but using N_3_β-CD (1 mM) instead of the β-CD dimer. After the reaction was complete, the slides were sonicated twice in DMSO (20 min) and once in milli-Q water and EtOH and finally dried under a stream of dry nitrogen.

**Binding assays in solution:** A 50 µM stock solution of 2,6-ANS in PBS (pH 7.4) was prepared and used to obtain 2 and 4 mM solutions of β-CD dimer and parent β-CD, respectively. The β-CD solutions were then diluted with the 2,6-ANS to generate a titration range with fixed 2,6-ANS concentrations. For each measurement, 5 scans (340–625 nm at λ_ex_ = 325 nm) where recorded with excitation and emission slits set to 5 nm and a PMT detector voltage of 400 V. The binding isotherms were fit by nonlinear regression using the one-site binding (hyperbola) model provided by GraphPad Prism software.

**Evaluation of inclusion complex by NMR spectroscopy:** A solution of 10 mM of β-CD dimer and 1 mM of 2,6-ANS in D_2_O (99.9%D) at pH 6.7 was used. Multiplicity-edited ^13^C HSQC [28–29] with matched sweep adiabiatic pulses [30] ^13^C HSQC–TOCSY (120 ms DIPSI2 mixing with γB_1_/2π = 9.6 kHz), ^13^C H2BC [31], DQF–COSY, 2D-NOESY (250 ms mixing time) and 2D-ROESY (250 ms mixing with γB_1_/2π = 5.9 kHz) spectra were used to obtain resonance assignment and determine the inclusion-complex structure.

**Evaluation of inclusion complex by TIRF spectroscopy:** 250 µL of 1 mM 2,6-ANS in PBS (pH 7.4) was injected into the flow cell mounted with either a bare quartz or a quartz slide grafted with PA or β-CD dimer. For each measurement, 10 scans were recorded (340–625 nm at λ_ex_ = 325) with excitation and emission slits set to 5 nm and a PMT detector voltage of 700 V.

## Supporting Information

File 1 ^1^H NMR and ^13^C HSQC spectra of β-CD dimer, ^13^C HSQC spectra of β-CD dimer in complex with 2,6-ANS and TIRF 2,6-ANS titration on bare quartz.
