# Supplementary material for: Synthesis and surface grafting of a β-cyclodextrin dimer facilitating cooperative inclusion of 2,6-ANS
Source: Beilstein J Org Chem. 2015 Apr 21;11:514–23. doi: 10.3762/bjoc.11.58 (PMC4419527; doi:10.3762/bjoc.11.58)
Supplement: File 1 — 1H NMR and 13C HSQC spectra of β-CD dimer, 13C HSQC spectra of β-CD dimer in complex with 2,6-ANS and TIRF 2,6-ANS titration on bare quartz. [file Beilstein_J_Org_Chem-11-514-s001.pdf]

Supporting Information

for

Synthesis and surface grafting of a β-cyclodextrin dimer facilitating cooperative inclusion of 2,6-ANS

Lars W. Städe[1], Thorbjørn T. Nielsen[1], Laurent Duroux[1], Reinhard Wimmer[1], Kyoko Shimizu[2] and Kim L. Larsen*[1]

Address: [1]Department of Biotechnology, Chemistry and Environmental Engineering, Aalborg University, Frederik Bajers Vej 7H, DK-9220 Aalborg East, Denmark and [2]Department of Chemistry, Aarhus University, Langelandsgade 140, DK-8000 Aarhus, Denmark

Email: Kim Lambertsen Larsen - kll@bio.aau.dk

*Corresponding author

^{1}H NMR and ^{13}C HSQC spectra of β-CD dimer, ^{13}C HSQC spectra of β-CD dimer in complex with 2,6-ANS and TIRF 2,6-ANS titration on bare quartz

NMR spectra

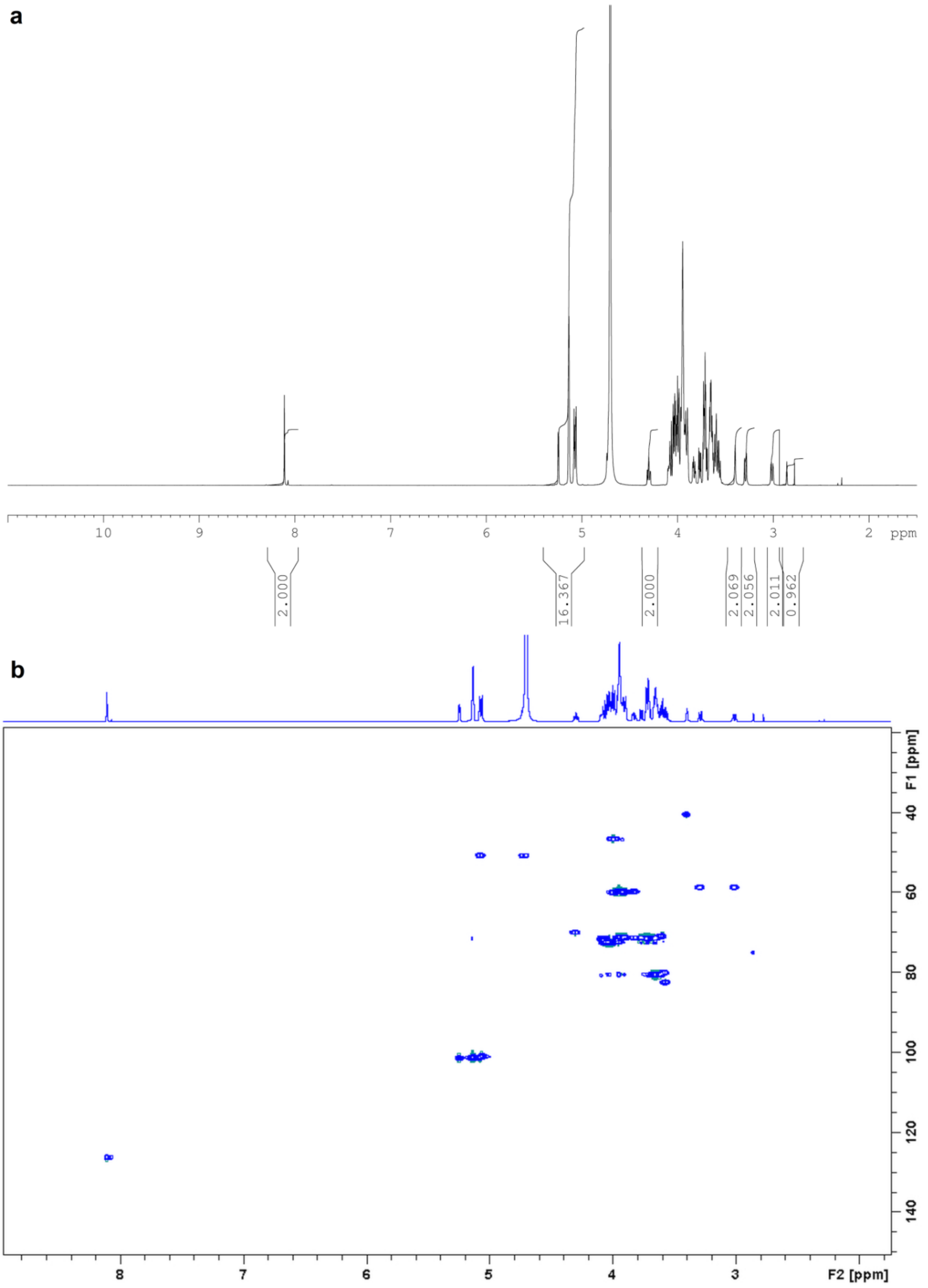

^{1}H NMR (a) and 13C HSQC (b) spectra of the β-CD dimer. Successful CuAAC is evident from the signal resonating at 8.1 ppm (triazole). Furthermore, a signal from the terminal alkyne is present at 2.9 ppm.

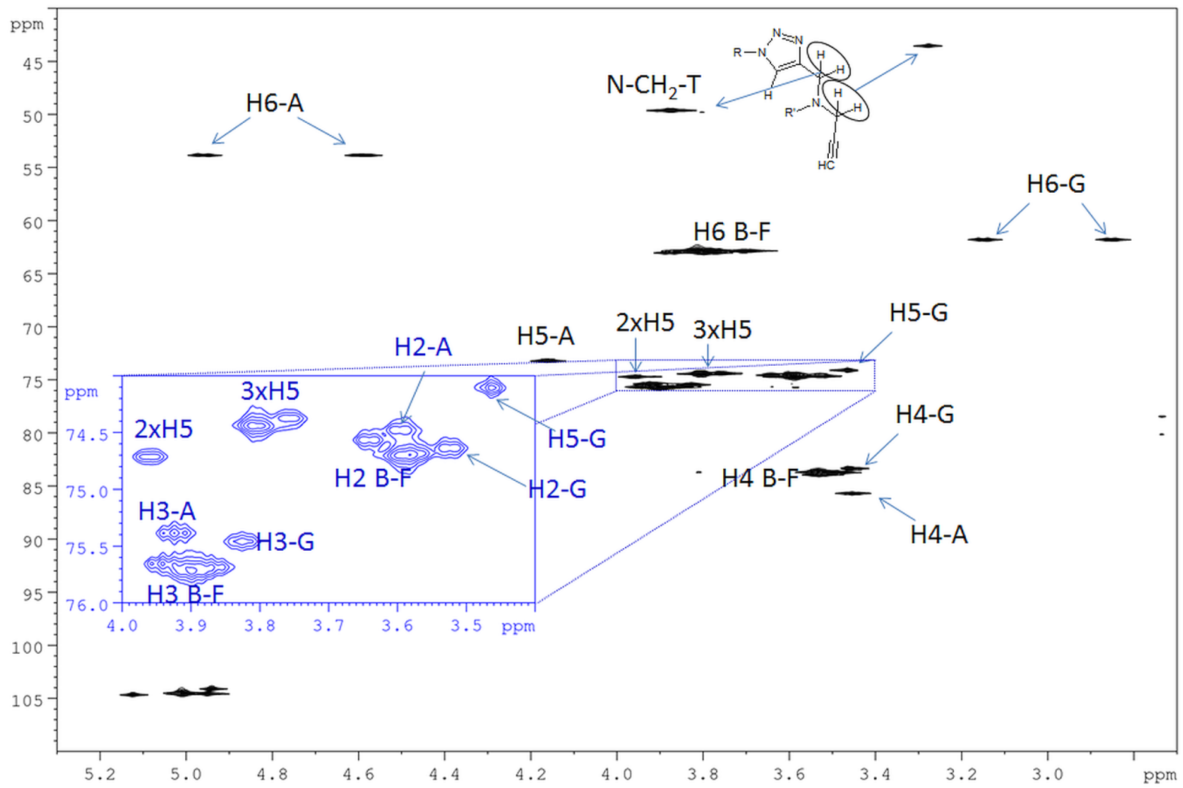

Region of a high-resolution ^{13}C HSQC spectrum of the β-CD dimer in complex with 2,6-ANS with the resonance assignment. The region containing H2, 3 and 5 is enlarged (in blue). Letters refer to the glucose monomer in the β-CD: “A” refers to the glucose unit carrying the triazole linker, while “G” refers to the glucose unit, whose C4 is glycosidically linked to the C1 of “A”.

Self-association/quenching of 2,6-ANS

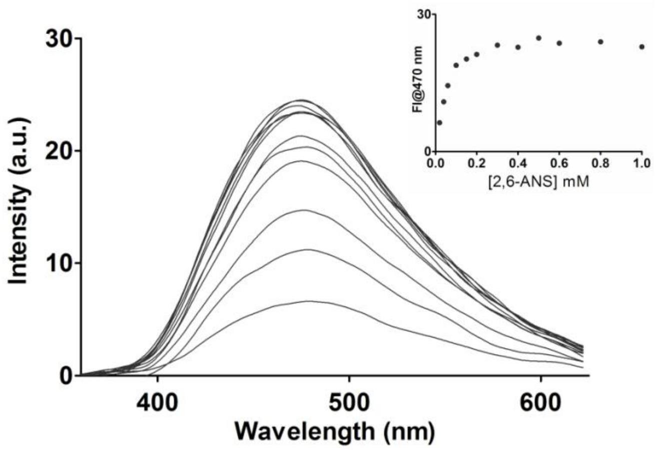

Titration series of 2,6-ANS (0.02–1 mM) recorded by TIRF spectroscopy on a clean and unmodified quartz slide.
